# Anatomic Pulmonary Lobectomy via Video-Assisted Thoracoscopic Surgery in an Adult Patient With Congenital Lobar Emphysema: A Case Report

**DOI:** 10.7759/cureus.114226

**Published:** 2026-08-09

**Authors:** Diego A Garrido Villamil, Andrea C Flórez Meneses, Carlos A Rodríguez Sabogal

**Affiliations:** 1 General Medicine, Universidad del Norte, Barranquilla, COL; 2 General Surgery, Hospital Militar Central, Bogotá D.C., COL; 3 Thoracic Surgery, Hospital Militar Central, Bogotá D.C., COL

**Keywords:** bronchomalacia, congenital lobar emphysema, congenital lobar overinflation, lung abscess, pulmonary lobectomy, rare diseases, video-assisted thoracoscopic surgery

## Abstract

Congenital lobar emphysema (CLE) is a congenital lung malformation characterized by progressive hyperinflation of a pulmonary lobe. We report the case of a 23-year-old male, an active-duty military service member with a history of recurrent pneumonias and lung abscesses since childhood, who presented with a new infectious episode. A chest computed tomography scan revealed an abscess in the right upper lobe, together with air trapping and overdistension of both the apical segment of the right upper lobe and the adjacent right middle lobe, raising suspicion for a congenital lung malformation. After achieving antibiotic control of the acute infection, a delayed right upper lobectomy was performed via video-assisted thoracoscopic surgery (VATS). The histopathological study confirmed focal bronchomalacia as the underlying finding. This case illustrates an atypical presentation of CLE in a young adult with severe infectious complications. A delayed VATS lobectomy, performed after control of the infectious process, achieved a good functional outcome. The postoperative course included a prolonged air leak that was managed conservatively on an outpatient basis and resolved without further morbidity. This case underscores the importance of a multidisciplinary approach and of individualized surgical planning in this rare disease.

## Introduction

Congenital lobar emphysema (CLE) is a rare entity within the spectrum of congenital lung malformations and is considered an orphan disease in Colombia [1]. Estimates derived largely from pediatric series suggest a prevalence of approximately 1/20,000 to 1/30,000 live births and a male predominance [2,3]. It is characterized by progressive hyperinflation of a pulmonary lobe secondary to air trapping caused by intrinsic (e.g., bronchomalacia) or extrinsic bronchial obstruction [4]. The left upper lobe is most frequently affected, followed by the right middle and right upper lobes, with the lower lobes involved less commonly [5]. Approximately 15% of cases are associated with congenital heart disease [3,6].

The diagnosis is usually established in the neonatal period or early childhood and is exceptional in adults. In this population, presentation is dominated by recurrent respiratory infections, although the condition is occasionally detected incidentally in asymptomatic adults [5,7,8]. We present a young adult in whom CLE remained undiagnosed until adulthood and manifested with a complicating lung abscess. The instructive aspects of this case are the delayed surgical strategy adopted after control of the infection, the discordance between the preoperative imaging and the intraoperative findings in the middle lobe, and the outpatient management of a postoperative prolonged air leak (PAL) with a Heimlich valve.

## Case presentation

A 23-year-old active-duty military service member presented with a history of recurrent pneumonia complicated by lung abscesses since his first year of life. One of these episodes required drainage at an outside institution, and all were treated with multiple courses of broad-spectrum antibiotics. He originated from a rural area of Southern Colombia. There was no known family history of congenital lung disease, and his psychosocial history was unremarkable apart from occasional alcohol use.

He was admitted to the emergency department with a 22-day history of productive cough, fever, hemoptysis, night sweats, and unintentional weight loss (12 kg over two months). On physical examination, he was hemodynamically stable, afebrile, and eupneic, without cyanosis, respiratory distress, or chest-wall deformity; oxygen saturation was 95% on room air. Decreased breath sounds were noted over the right hemithorax, and he reported no chest pain.

**Figure 1:**
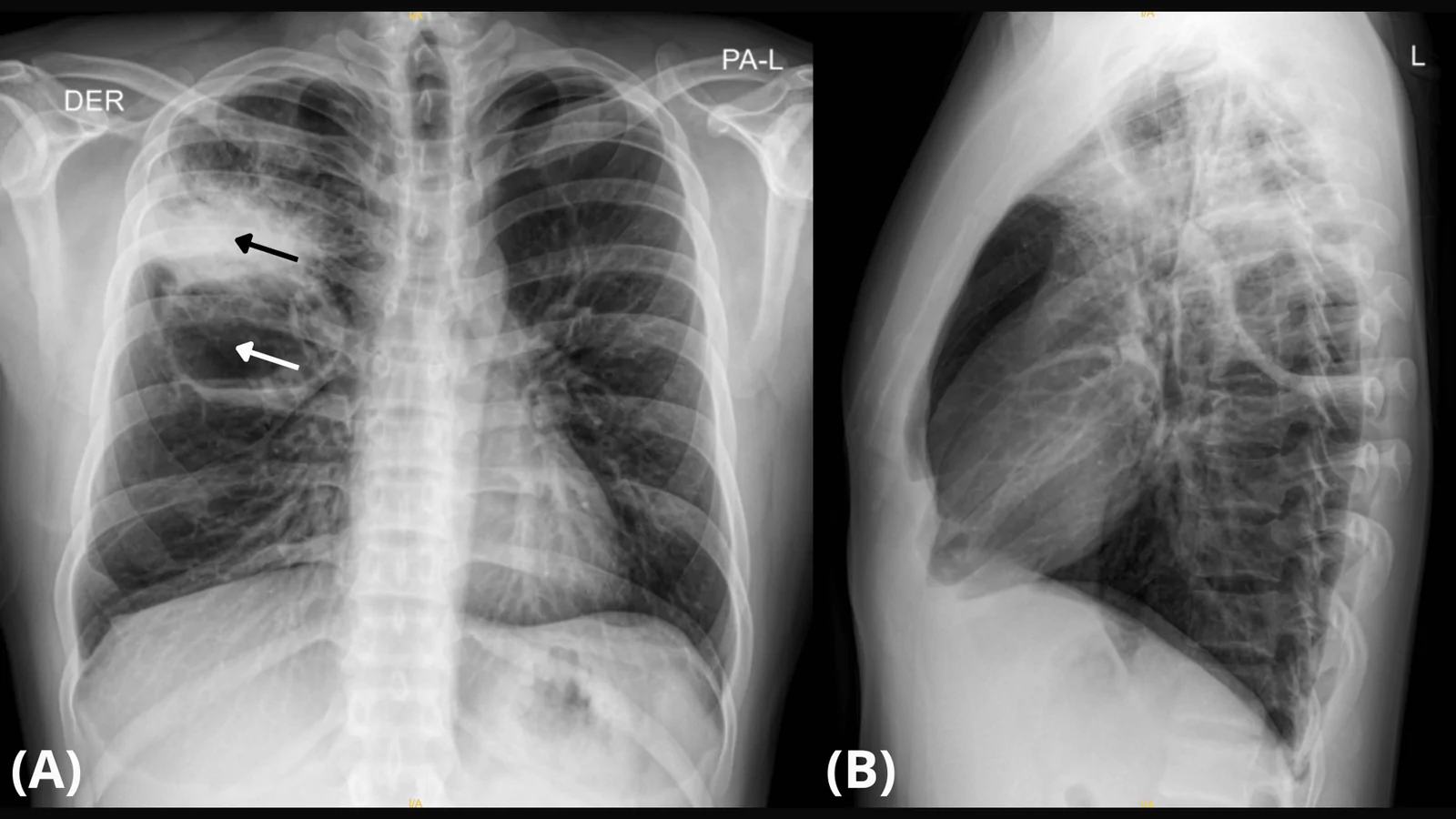
Posteroanterior and lateral chest radiographs on admission. (A) Posteroanterior chest radiograph showing right upper lobe consolidation (black arrow) overlying a radiolucent, cystic-appearing lesion (white arrow). (B) Corresponding lateral chest radiograph.

Given the initial suspicion of pulmonary tuberculosis, a chest radiograph was obtained (Figure 1), revealing an irregular opacity in the right upper lobe with an underlying radiolucent area suggestive of a cystic lesion. Laboratory studies on admission showed reactive thrombocytosis (608 × 10³/µL), neutrophilia (74.8%), a markedly elevated high-sensitivity C-reactive protein (6.85 mg/dL), and an elevated D-dimer of 2.42 µg/mL, while renal and hepatic function remained within normal limits. Arterial blood gas analysis indicated a mild respiratory alkalosis (pCO₂, 33 mmHg), with a PaO₂ of 91 mmHg and an oxygen saturation of 97.7%, without significant resting hypoxemia.

Microbiological studies for tuberculosis were negative, including two serial sputum smears, GeneXpert MTB/RIF (*Mycobacterium tuberculosis*/rifampicin resistance) assays performed on both sputum and bronchoalveolar lavage (BAL) specimens, and liquid mycobacterial cultures of sputum and BAL, which remained negative after six weeks of incubation. Routine bacterial culture of BAL was negative after 72 hours, and fungal culture remained negative after four weeks. Serum immunoglobulin G was mildly elevated (2,051 mg/dL; reference range: 610-1,616 mg/dL), a nonspecific finding that may accompany chronic or recurrent infection. Laboratory findings are summarized in Table 1.

**Table 1:** Laboratory and microbiological findings. Day 1 corresponds to the day of hospital admission. Arterial blood gases were obtained on room air. Microbiological cultures are dated according to the day the final result was reported. *Values marked with an asterisk are outside the reference range. AFB: acid-fast bacilli; ALT: alanine aminotransferase; AST: aspartate aminotransferase; BAL: bronchoalveolar lavage; CRP: C-reactive protein; MTB: *Mycobacterium tuberculosis*; MTB/RIF: *Mycobacterium tuberculosis*/rifampicin resistance.

Test	Result	Reference range	Day
Admission			
Leukocytes	9.85 × 10³/mm³	4.5–11.3 × 10³/mm³	Day 1
Neutrophils	74.8% *	50–65%	Day 1
Platelets	608 × 10³/µL *	150–450 × 10³/µL	Day 1
Hemoglobin	13.6 g/dL	12.1–16.6 g/dL	Day 1
High-sensitivity CRP	6.85 mg/dL *	0–0.5 mg/dL	Day 1
D-dimer	2.42 µg/mL *	0–0.50 µg/mL	Day 1
pH	7.430	7.35–7.45	Day 1
pCO₂	33.0 mmHg *	35–48 mmHg	Day 1
PaO₂	91.0 mmHg	83–108 mmHg	Day 1
SaO₂	97.7%	94–98%	Day 1
HCO₃⁻	21.9 mmol/L	21–28 mmol/L	Day 1
Blood urea nitrogen	6.7 mg/dL	6–20 mg/dL	Day 1
Serum creatinine	0.78 mg/dL	0.67–1.17 mg/dL	Day 1
ALT	16 U/L	0–41 U/L	Day 2
AST	20 U/L	0–40 U/L	Day 2
Follow-up			
Leukocytes	6.58 × 10³/mm³	4.5–11.3 × 10³/mm³	Day 10
Neutrophils	56.0%	50–65%	Day 10
Platelets	368 × 10³/µL	150–450 × 10³/µL	Day 10
Hemoglobin	14.1 g/dL	12.1–16.6 g/dL	Day 10
Blood urea nitrogen	10.1 mg/dL	6–20 mg/dL	Day 10
Serum creatinine	1.04 mg/dL	0.67–1.17 mg/dL	Day 10
Immunology			
Immunoglobulin G (IgG)	2,051 mg/dL *	610–1,616 mg/dL	Day 6
Microbiology			
AFB smear microscopy, sputum (2 samples)	Negative	Not applicable	Day 2
GeneXpert MTB/RIF, sputum	MTB not detected	Not applicable	Day 2
Gram stain, BAL	No microorganisms	Not applicable	Day 3
Ziehl–Neelsen (AFB), BAL	Negative	Not applicable	Day 4
GeneXpert MTB/RIF, BAL	MTB not detected	Not applicable	Day 4
Routine bacterial culture, BAL	Negative at 72 hours	Not applicable	Day 6
Fungal culture, BAL	Negative at 4 weeks	Not applicable	Day 37
Mycobacterial culture, sputum	Negative at 42 days	Not applicable	Day 53
Mycobacterial culture, BAL	Negative at 42 days	Not applicable	Day 53

**Figure 2:**
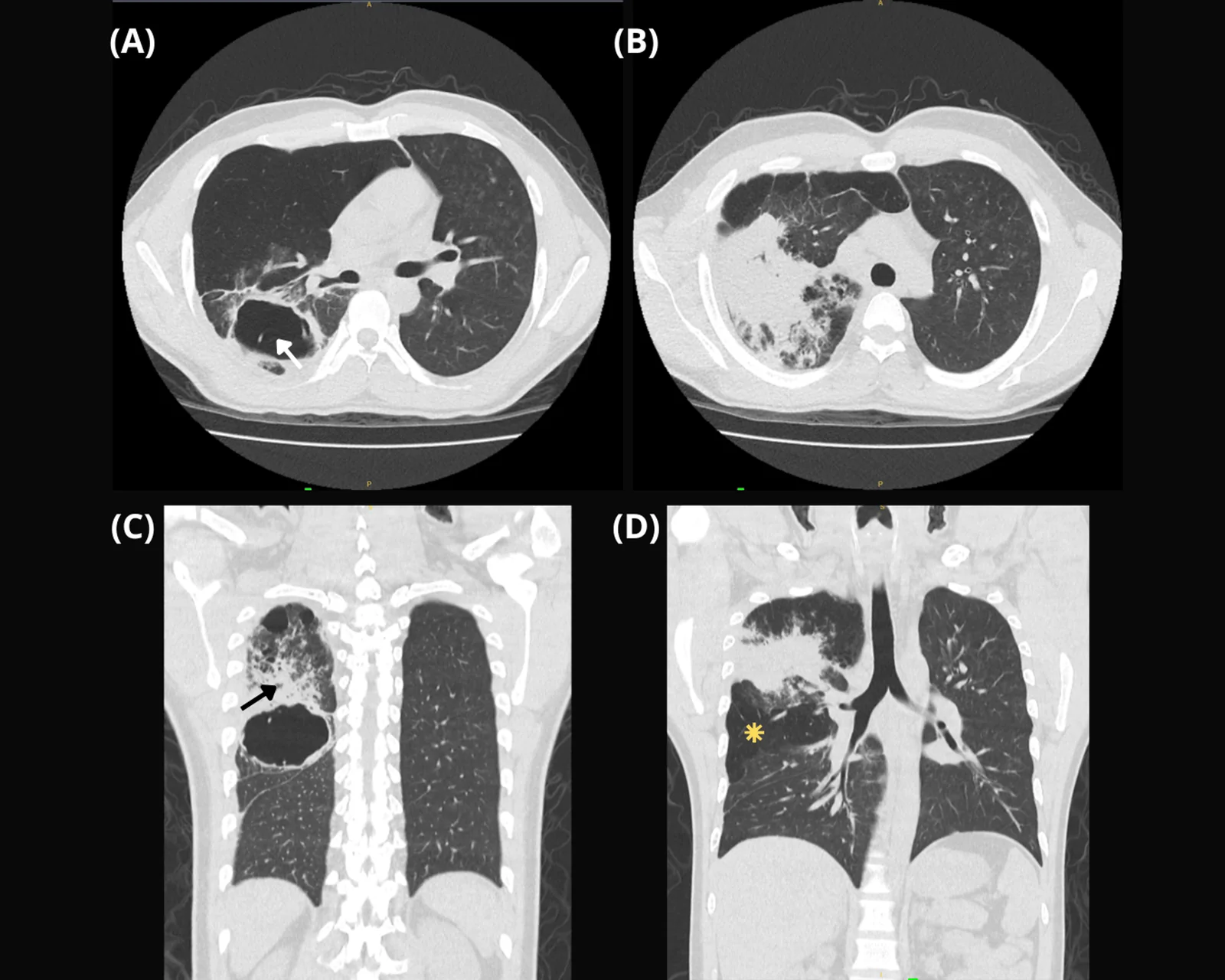
Contrast-enhanced chest computed tomography on admission. Axial (A, B) and coronal (C, D) images demonstrate right upper lobe consolidation (black arrow) associated with a thin-walled cavitary lesion containing an air-fluid level (white arrow), consistent with a lung abscess, together with hyperinflation of the adjacent right middle lobe (*).

A contrast-enhanced chest computed tomography (CECT) scan (Figure 2) depicted right upper lobe consolidation with air bronchograms and a thin-walled, subpleural cavitary lesion measuring 59 × 50 × 74 mm and containing an air-fluid level, consistent with a lung abscess. Severe air trapping produced overdistension of the apical segment of the right upper lobe and of the adjacent right middle lobe, without endobronchial obstruction or bronchiectasis.

Empirical antimicrobial therapy was started with intravenous piperacillin-tazobactam (4.5 g every six hours) and vancomycin (1 g every 12 hours), with a favorable clinical response. After infectious disease evaluation and in the absence of an identified pathogen, vancomycin was discontinued, and piperacillin-tazobactam was continued as monotherapy (as an extended four-hour infusion every eight hours) to complete a 21-day course. Given the history of recurrent pulmonary infections and imaging findings suggestive of CLE, the patient was evaluated by the thoracic surgery team. Although resection was indicated, lobectomy was intentionally deferred until control of the acute infectious process, in keeping with our institutional strategy to reduce the risk of postoperative bronchopleural fistula. The lobectomy was ultimately performed approximately one month after admission, nine days after completion of the antibiotic course.

Preoperative spirometry showed preserved lung function, with a forced vital capacity (FVC) of 4.50 L (84% of predicted), a forced expiratory volume in one second (FEV₁) of 3.60 L (80% of predicted), and an FEV₁/FVC ratio of 80.07% (94% of predicted). Test quality was graded A according to the 2019 American Thoracic Society/European Respiratory Society standards [9]. This non-obstructive pattern, together with a peak expiratory flow (PEF) of 10.92 L/s (108% of predicted), confirmed adequate pulmonary functional reserve for the planned resection.

**Figure 3:**
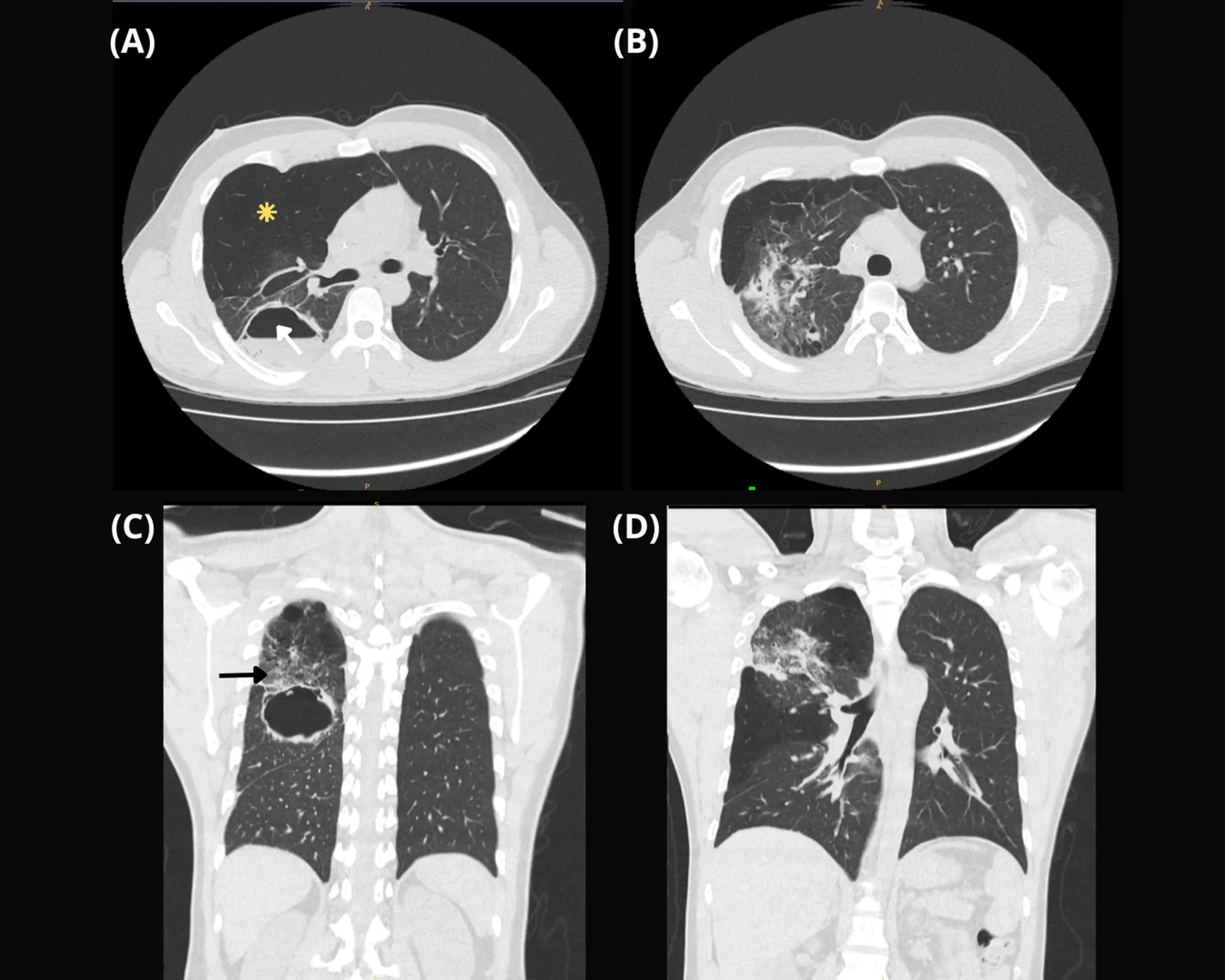
Follow-up contrast-enhanced chest computed tomography after completion of antibiotic therapy. Axial (A, B) and coronal (C, D) images demonstrate partial resolution of the right upper lobe consolidation (black arrow), persistence of a thin-walled cavitary lesion (white arrow), and persistent hyperinflation of the right middle lobe (*).

**Figure 4:**
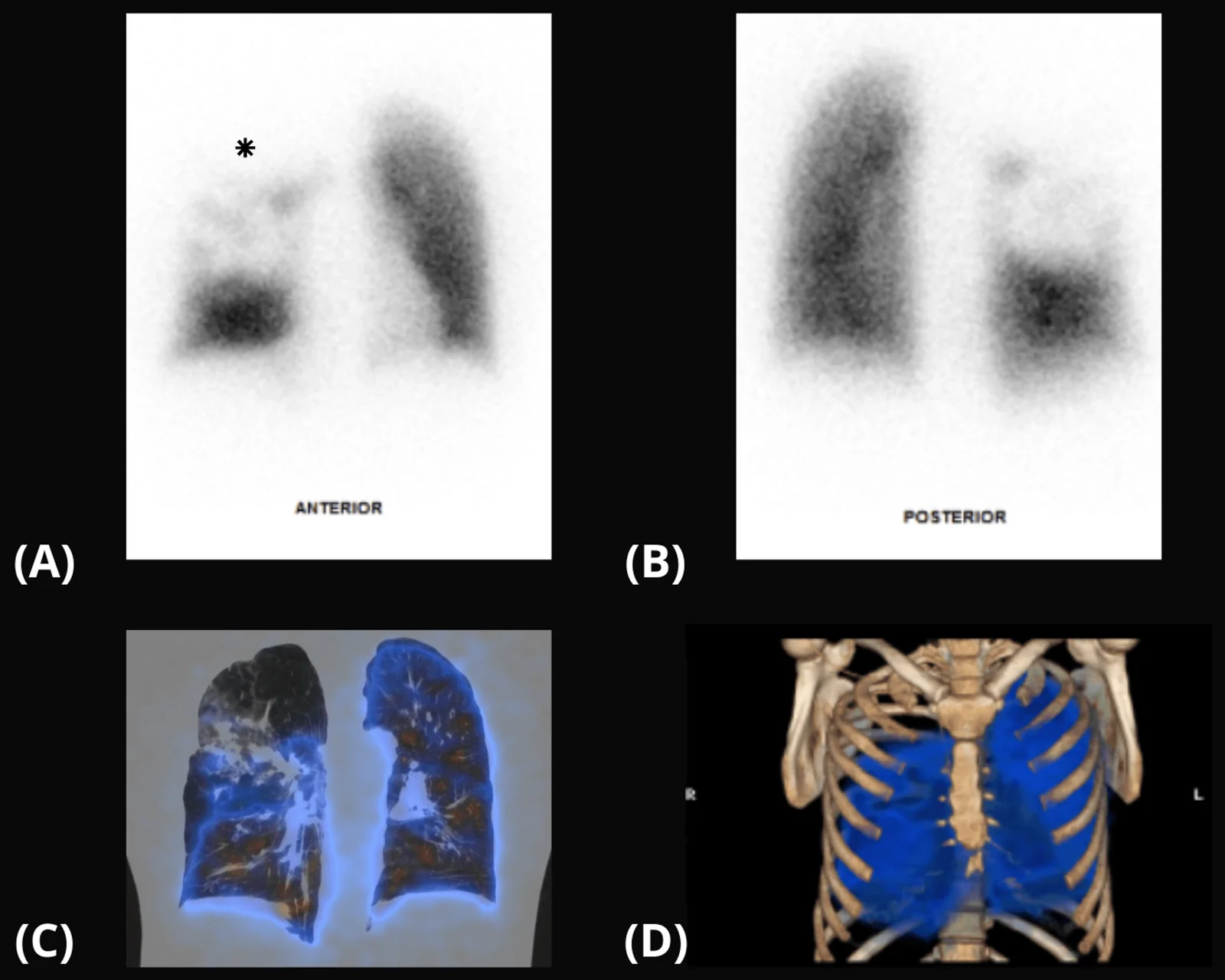
Pulmonary ventilation/perfusion imaging. Anterior (A) and posterior (B) planar perfusion images, together with functional perfusion displays (C, D), demonstrate severe perfusion defects involving the right upper and middle lobes (*), with preserved perfusion of the left lung.

Following completion of antibiotic therapy, repeat chest CT (Figure 3) revealed partial resolution of the consolidation but persistent cavitary disease and hyperinflation of the middle lobe. A ventilation/perfusion lung scan (Figure 4) disclosed severe perfusion defects in the right upper and middle lobes.

Given the diagnosis of CLE complicated by a chronic lung abscess and preserved pulmonary function, anatomic resection was scheduled by video-assisted thoracoscopic surgery (VATS). Because preoperative imaging had shown overdistension of both the apical segment of the right upper lobe and the right middle lobe, resection of both lobes had initially been considered. Intraoperatively, multiple visceroparietal and visceropericardial adhesions were found, predominantly affecting the right upper lobe, which had a markedly emphysematous appearance, and an abscess was contained within that lobe. In contrast to its hyperinflated appearance on imaging, the right middle lobe was atrophic and adherent to the upper lobe, with otherwise grossly normal parenchyma, and it was therefore preserved.

The procedure was completed by conventional two-port VATS, using a camera port in the seventh intercostal space (midaxillary line) and a utility port in the fourth intercostal space (anterior axillary line) with a wound protector. Following adhesiolysis, a parietal pleurectomy was performed, and the superior pulmonary artery, the superior pulmonary vein, and the upper lobe bronchus were individually divided with mechanical staplers; the fissure was then completed, and an anatomic right upper lobectomy with level-10 mediastinal lymph node dissection was carried out. Intraoperative testing confirmed adequate reexpansion of the middle and lower lobes. Operative time was approximately three hours and 20 minutes, intraoperative bleeding was minimal, and there were no intraoperative complications or conversion to thoracotomy.

**Figure 5:**
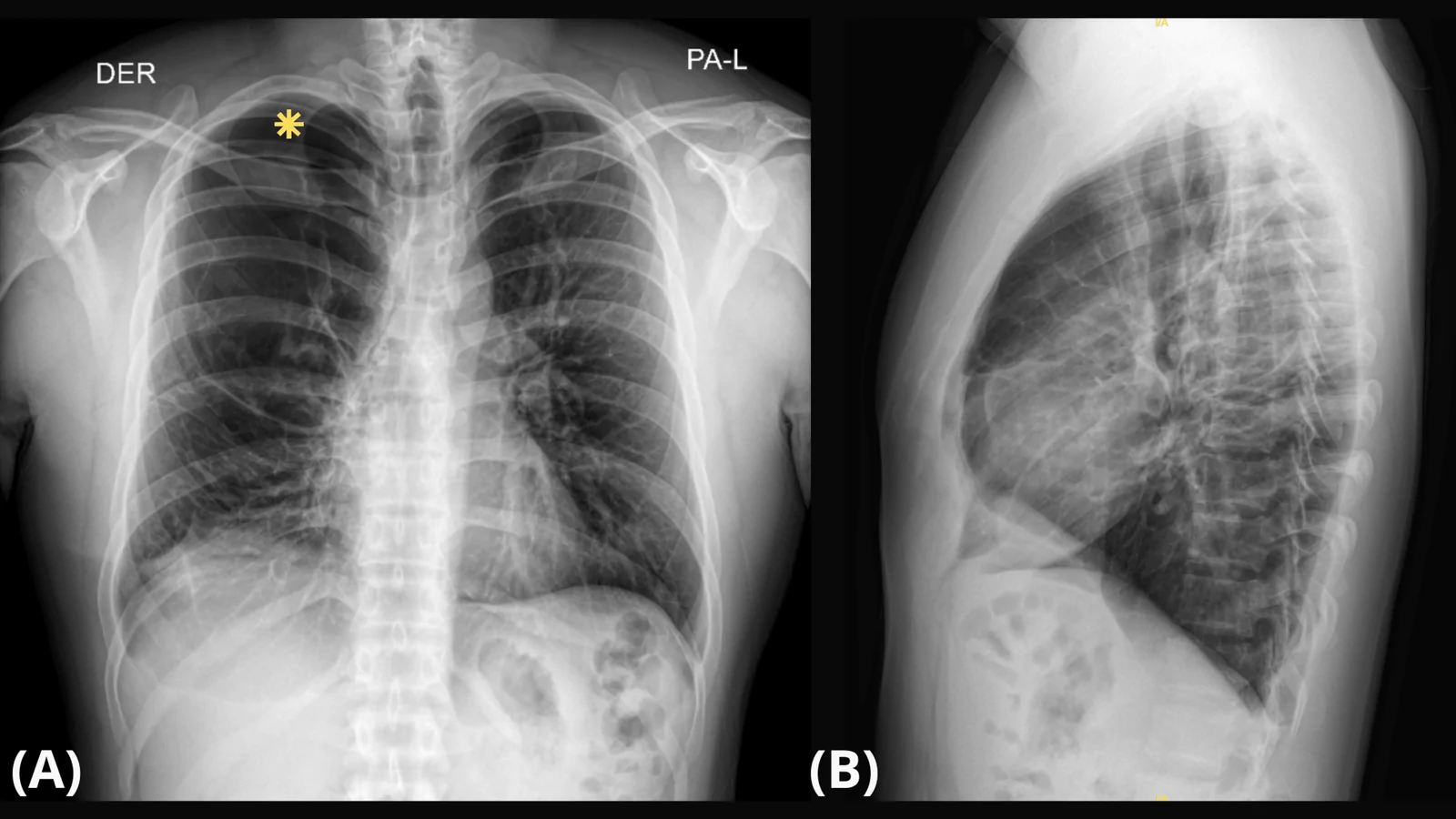
Follow-up chest radiographs seven months after surgery. (A) Posteroanterior and (B) lateral chest radiographs obtained seven months after the right upper lobectomy show no consolidation, normal pulmonary vascularity, and free costophrenic angles. A small residual apical space is seen on the right (*), an expected finding after upper lobectomy, in an asymptomatic patient.

The postoperative hospital stay lasted 18 days and included pulmonary rehabilitation and chest tube drainage under continuous suction. Recovery was extended by a PAL, which was managed on an outpatient basis with a Heimlich valve after discharge. One month after surgery, the chest tube was removed once imaging confirmed complete lung reexpansion, and the patient returned to active military duty without further complications. On postoperative imaging, the changes previously noted in the right middle lobe were attributed to reexpansion pulmonary edema and compensatory hyperinflation, with no residual structural abnormality identified. From the patient's perspective, he reported a subjective return to his prior functional status and was able to resume his active military duties without limitation. At seven months after surgery, he remained asymptomatic, and follow-up chest radiographs showed no consolidation, with a small expected residual apical space on the right (Figure 5).

Histopathological examination of the surgical specimen confirmed the clinical and radiological diagnosis. The right upper lobe displayed architectural distortion characterized by widening of the alveolar septa, with focal interstitial fibrosis and a chronic lymphohistioplasmacytic inflammatory infiltrate. The alveolar spaces were dilated and contained abundant foamy histiocytes, lymphocytes, and occasional foreign-body giant cells, in keeping with chronic inflammatory change secondary to recurrent infection. The bronchial wall exhibited fibrosis with focal destruction of the cartilaginous plates, consistent with focal bronchomalacia.

Additional findings included fibroblastic polyps in the peripheral airways, pleural fibrosis with neovascularization, and adjacent lung parenchyma with preserved overall architecture, mildly widened septa, mild mononuclear inflammatory infiltrates, subpleural emphysematous change, and bleb formation. The resected lymph node revealed sinus histiocytosis with anthracotic pigment deposition and no evidence of malignancy. Periodic acid-Schiff and Gomori methenamine silver stains were negative for fungal organisms, Ziehl-Neelsen staining was negative for acid-fast bacilli, and Masson trichrome staining highlighted focal interstitial fibrosis without dense fibrotic remodeling. Taken together, the histopathological findings corroborated chronic inflammatory changes with focal interstitial fibrosis, emphysema, and focal bronchomalacia, without granulomatous inflammation, diffuse fibrosis, or malignancy.

Written informed consent for the publication of this case and the accompanying images was obtained from the patient.

## Discussion

CLE, also known as congenital lobar overinflation, arises from a focal abnormality of the bronchial cartilage that creates a check-valve mechanism and leads to progressive air trapping [10,11]. Although most cases are recognized in the neonatal period, presentation in adulthood is exceptional and poses a diagnostic challenge, since it can be mistaken for acquired entities such as pneumothorax, emphysematous bullae, bronchiectasis, or even lung neoplasms [11,12]. In this context, CT is the most reliable tool for distinguishing CLE from other congenital and acquired anomalies, revealing lobar hyperinflation, mediastinal shift, and preservation of the vascular architecture [10,11]. The present case reinforces the importance of including CLE in the differential diagnosis of adults with chronic or recurrent pulmonary infections.

Histopathologically, CLE is characterized by alveolar hyperinflation without parenchymal destruction, thinned alveolar walls, and absence or hypoplasia of the bronchial cartilage. These features distinguish it from acquired emphysematous processes, in which there is irreversible loss of the alveolar septa. In adults, histological confirmation after surgical resection remains valuable, because the clinical and imaging presentation can overlap with other congenital malformations and with acquired conditions such as chronic bronchiectasis. A review of published cases shows that misdiagnosis is frequent and that anatomic resection not only confirms the congenital nature of the lesion but also prevents potentially fatal complications, such as tension pneumothorax and recurrent infection [12]. In our patient, the surgical specimen showed focal rather than diffuse cartilage destruction with focal bronchomalacia and emphysema, and the adjacent parenchyma was largely preserved, a pattern more consistent with an underlying congenital structural defect than with purely acquired, infection-related cartilage damage. Nonetheless, given the years of recurrent infection, a superimposed acquired component cannot be entirely excluded, and this is an inherent limitation of histological interpretation in long-standing disease. Alternative diagnoses (including congenital pulmonary airway malformation, bronchial atresia, an infected bulla, and a post-infectious pneumatocele) were considered. Notably, the histopathological report also raised intralobar pulmonary sequestration as a morphological differential and recommended clinical, radiological, and intraoperative correlation. CECT described the mediastinal great vessels as normal but did not specifically characterize the arterial supply of the lesion, and no anomalous systemic feeding artery was encountered intraoperatively; a dedicated CT-angiographic assessment of the systemic vasculature was not performed, and intralobar sequestration therefore could not be formally excluded on imaging. The recurrent course since infancy, the pattern of lobar overdistension, and the histological findings favored CLE, but we acknowledge this diagnostic limitation.

Although lobectomy is the treatment of choice for symptomatic CLE, the decision to operate and the optimal timing must be individualized. When active infection is present, as in the case reported here, we consider it prudent to defer surgery until medical control of the acute process is achieved, with the aim of reducing perioperative risks, chiefly bronchopleural fistula [13]. The delayed VATS lobectomy performed in this patient supports that strategy. Previous studies suggest that patients who undergo lobectomy tend to be more symptomatic and are diagnosed earlier than those managed conservatively [14]; these series, however, may underestimate the proportion of patients with mild or asymptomatic CLE who never require surgery. Indeed, selected patients with symptomatic CLE managed conservatively have shown good short- and long-term outcomes, non-inferior to those of surgically resected cases [15], which reinforces that the indication for and timing of surgery must be individualized rather than applied uniformly.

Chronic inflammatory processes, such as recurrent lung abscesses, add further complexity to the surgical management of CLE in adults. Persistent inflammation promotes pleural adhesions and parenchymal fragility, raising the risk of a PAL after VATS lobectomy. The literature has consistently identified several independent risk factors for PAL, including chronic obstructive pulmonary disease (COPD), reduced lung function (low FEV₁, a decreased FEV₁/FVC ratio, and a low diffusing capacity for carbon monoxide), a history of smoking, male sex, low body mass index, pleural adhesions, incomplete fissures, upper lobe resection, and more complex procedures with prolonged operative times and intraoperative bleeding [16-18].

In our patient, preoperative lung function was preserved, and there was no history of COPD or smoking, which excluded several of the most common risk factors. Nonetheless, the extensive pleural adhesions and the upper lobe resection remained relevant predisposing conditions. The interplay between the underlying congenital disease and these acquired inflammatory changes accounts for the vulnerability of such patients and underscores the need to weigh anatomical and functional characteristics together during surgical planning [16,17].

PAL, defined as an air leak persisting for more than seven days, is a recognized complication of lobectomy, particularly in lungs with emphysematous disease or prior infection. Outpatient management with a Heimlich valve, as used in this patient, is a safe and effective approach that enables home-based recovery and shortens hospital stay [18-20]. In this patient, although the outcome was ultimately favorable, the procedure entailed meaningful morbidity, including an 18-day postoperative hospital stay and a PAL that required outpatient chest drainage for one month; this morbidity should be weighed when counseling similar patients.

This case also highlights persistent gaps in the literature on the optimal management of CLE in adults. Because symptomatic localized CLE is conventionally treated by lobectomy, the timing of surgery after control of infection is best regarded as an individualized clinical decision rather than a novel intervention. Further reports are needed to define the optimal timing of surgery in adults presenting with infectious complications, and a multidisciplinary approach remains fundamental to decision-making.

Certain limitations of this report should be acknowledged. The observations described here should be interpreted within the context of this study design. The postoperative follow-up extended to seven months, documenting a favorable short- to medium-term course, but does not inform on long-term outcomes. Genetic testing and quantitative split lung-perfusion studies were beyond the scope of the diagnostic work-up, and, although the imaging and intraoperative findings supported CLE, a dedicated CT-angiographic evaluation of the systemic arterial supply was not part of the protocol; intralobar pulmonary sequestration, raised as a morphological differential in the pathology report, therefore remained a theoretical consideration.

## Conclusions

CLE can present atypically in adults, with severe infectious complications (including recurrent lung abscesses) that substantially affect quality of life. This case illustrates that a late diagnosis does not preclude definitive surgical management and that, in an adult with a clearly identified diseased lobe, anatomic VATS lobectomy can achieve a good functional outcome after control of an acute infection, although this was attained at the cost of meaningful postoperative morbidity in the form of a PAL. Such complications can nonetheless be managed in the outpatient setting with a satisfactory final result.

This report underscores the need for a high index of suspicion for congenital pulmonary malformations in young adults with recurrent, localized pulmonary infection, and it supports an individualized, multidisciplinary approach to the indication for and timing of surgery once the diseased lobe is clearly identified. Further reports and prospective data are warranted to refine the management of CLE in adults and to establish higher-quality, evidence-based recommendations.
